# Life History Parameters of the Invasive Cotton Mealybug *Phenacoccus solenopsis* on Tomato at Four Constant Temperatures

**DOI:** 10.3390/insects16010016

**Published:** 2024-12-27

**Authors:** Ahlem Harbi, Khaled Abbes, Brahim Chermiti, Pompeo Suma

**Affiliations:** 1 Department of Biological Sciences and Plant Protection, High Agronomic Institute of Chott-Mariem, University of Sousse, Chott-Mariem 4042, Tunisia; abbes.kaled@live.fr (K.A.); chermiti54@yahoo.fr (B.C.); 2 Laboratory of Agrobiodiversity and Ecotoxicology LR21AGR02, University of Sousse, Sousse 4000, Tunisia; 3 Department of Agriculture, Food and Environments, University of Catania, I-95123 Catania, Italy; suma@unict.it

**Keywords:** population dynamics, population projection, pest fitness, life tables, integrated pest management

## Abstract

The cotton mealybug, *Phenacoccus solenopsis* Tinsley (Hemiptera: Pseudococcidae), is an invasive polyphagous pest that has been reported in several tomato-producing Mediterranean countries. However, information regarding the impact of temperature variations on its potential damage and population dynamics on this crop is limited. The effect of four temperatures (20 ± 1 °C, 25 ± 1 °C, 30 ± 1 °C and 35 ± 1 °C) on the development, reproduction, and population growth parameters of *P. solenopsis* on tomatoes under controlled laboratory conditions was investigated using age-stage two-sex life tables. The increase in temperature caused a significant decrease in the developmental periods of all instars except eggs. The shortest durations of the life cycle (29.58 ± 0.28 days for females and 13.91 ± 0.25 days for males), the adult preoviposition period (APOP), and the total preoviposition period (TPOP) (APOP: 7.78 ± 0.09 days and TPOP: 18.33 ± 0.13 days) were obtained at 35 ± 1 °C. Fecundity varied with temperature, and the highest value was recorded at 30 ± 1 °C (183.29 ± 7.13 eggs/female). The highest average net reproduction rate (*R*_0_) (154.24 ± 14.681 offspring/female), intrinsic rate of increase (*r*) (0.222 ± 0.0036 d^−1^), and finite rate of increase (*λ*) (1.248 ± 0.00495 d^−1^) were observed at 35 ± 1 °C. A simulation of population increase and structure under different temperatures over a period of 90 days revealed that the greatest expected population size was at 35 ± 1 °C, with the completion of four overlapping generations. The data from this study provide valuable information for adapted pest management approaches against *P. solenopsis* on tomato crops.

## Simple Summary

This study explores the effect of temperature on the invasive cotton mealybug, *Phenacoccus Solenopsis*, a new pest of tomatoes in Mediterranean countries. We investigated how temperature influences its damage and population growth under controlled laboratory conditions using age-stage two-sex life tables. We found that as temperature increased, the developmental time of the mealybug’s life stages decreased significantly. The shortest life cycle durations and preoviposition periods were observed at 35 °C. Fecundity peaked at 30 °C while the highest reproductive rates and population growth parameters were recorded at 35 °C. Simulation of population growth over 90 days at these temperatures indicated that the largest mealybug population is produced at 35 °C with four overlapping generations. These findings are crucial for developing temperature-sensitive pest management strategies to suppress *P. solenopsis* populations effectively in tomato crops across invaded regions.

## 1. Introduction

Invasive species pose the most significant global threat to ecosystems, causing direct and indirect ecologic and economic damage [1,2]. Globalization, international trade of seeds, seedlings, and agricultural products, modern agricultural practices such as monoculture, massive use of chemical inputs, and climate change have caused a significant increase in biological invasion phenomena in agricultural ecosystems, menacing food security and production sustainability [3]. The introduction of alien agricultural insect pests is accompanied by exceptional countermeasures, including massive insecticide sprays, leading, in many cases, to the disruption of integrated pest management (IPM) programs and functional biodiversity.

The cotton mealybug *Phenacoccus solenopsis* Tinsley (Hemiptera: Pseudococcidae) is an economic polyphagous pest native to North America. In 2005, it invaded Asian industrial cotton cultivations and caused massive economic losses, especially in India, China, and Pakistan [4]. In 2021, *P. solenopsis* has been reported to infest ornamental plants and Solanaceous horticultural crops in several Mediterranean countries [5,6,7,8]. It feeds on the areal parts of plants by sucking sap, producing copious amounts of honeydew, and causing sooty mold, which interferes with photosynthesis and reduces yields. Apart from cotton, its primary host plant, many Solanaceous crops have become common hosts for this pest in its new distribution areas, such as Algeria, Israel, Egypt, Italy, and Tunisia. In countries where cotton cultivation is not frequent, the pest can shift to Solanaceous and potentially cause economic losses [9].

Tomatoes are an important crop worldwide, especially in the Mediterranean Basin. Italy accounted for close to two-fifths (39.8%) of the EU’s harvested production of tomatoes in 2022, and in North Africa, it is widely cultivated in Morocco and Tunisia [10]. In previous work, we demonstrated that tomato is a very suitable host crop for *P. solenopsis* [9], particularly in greenhouses, where optimal temperature range and high host density provide ideal conditions for pest outbreaks. As ectotherms, temperature has a great impact on the biology of insect pests, influencing their behavior, development, reproduction, and senescence [11]. Understanding the role of temperature on life traits is particularly important in the case of invasive pests because they often encounter different climatic conditions that directly impact their ability to establish and spread in non-native regions. Moreover, temperature affects the synchronization of life cycles between invasive pests and their host plants, which can affect their success in new environments [12,13]. Thus, understanding the effect of temperature on this invasive insect when infesting tomatoes is crucial for the development of IPM schemes [14,15,16] by providing valuable information about its phenology, population dynamics, and structure and planning of insecticide treatments and release of natural enemies [17]. In this context, studying life history traits and population variations through life table analysis is essential for optimized pest control. It provides data on insect biology and reproduction parameters that can be used to predict potential damage under various environmental conditions [18].

Owing to the invasion and the great economic impact on the Asian industrial cotton cultivations [19] [Liu et al. 2024], most studies on the biotic potential of *P. solenopsis* focused on cotton as a host plant [20]. However, research on its life history and damage potential to tomatoes, considering the effect of temperature in the context of global warming, is scarce. Considering the recent invasion of several Mediterranean countries by this pest where tomato is a popular crop, and to fill this gap, we studied the life table parameters and population projection of *P. solenopsis* on tomatoes at different temperature regimes using the age-stage two-sex life table method.

## 2. Materials and Methods

**Plants.** The tomato plants (commercial variety ‘Dorra’, ‘Planète verte’, Nabeul, Tunisia) used in the experiments were grown in April 2024 from seeds in plastic pots (33 cm × 25 cm × 13 cm) filled with peat substrate, watered when needed, and maintained in a greenhouse under natural conditions without chemicals inputs until reaching a 10–15 cm height.

**Insect stock.** *Phenacoccus solenopsis* individuals were obtained from a laboratory rearing maintained in a climatic chamber at 25 ± 2 °C, 60 ± 10% RH, and a 16:8 h (L:D) photoperiod. The colony was established starting from specimens collected on *Lantana camara* L. (Verbenaceae) in Tunis, Tunisia, in the spring of 2022. The mealybug was reared for ten generations on tomatoes before its use in the experiment to avoid possible effects of host shifting on studied parameters [9].

**Experimental protocol.** For each studied temperature regime (20 ± 1 °C, 25 ± 1 °C, 30 ± 1 °C, and 35 ± 1 °C), 75 tomato plants were individually transplanted into plastic cups of a 200 mL volume filled with peat substrate. Peer cohorts of *P. solenopsis* were formed and used to collect eggs. Each freshly laid eggs (<1 h) were placed on an apical tomato leaf using a fine, soft paintbrush, and the cups were sealed with a piece of mousseline cloth held with a rubber band to avoid the escape of insects. Infested tomato plants, each bearing one egg, were subsequently incubated in climatic cabinets (Scimmit, Shanghai Scimmit Technology, Shanghai, China), previously set at corresponding considered temperatures with 60 ± 5% relative humidity and a 16:8 h (L:D) photoperiod. All plants were watered when needed. The complete life cycle of each individual was monitored daily until its death using a binocular microscope (Leica MZ8, Leica Microsystems, Wetzlar, Germany). The change of the nymphal stage was recorded when molt exuviae were observed. Newly emerged adults were used to form couples, and the following parameters were registered: egg incubation period, duration of each developmental stage, adult preoviposition period (APOP), total preoviposition period (TPOP), oviposition days, fecundity, adult sex, and longevity.

**Demographic analyses.** Raw data on the development and reproduction of the mealybug on tomatoes under each temperature regime were used to construct two-sex life tables according to Chi and Liu [21] and Huang and Chi [22]. We calculated the age-stage-specific survival rate (s*_xj_*), age-specific survival rate (*l_x_*), age-stage-specific life expectancy (*e_xj_*), age-stage-specific fecundity (*f_x_*), reproductive value (*v_xj_*), and age-stage fecundity (*m_x_*). The net reproductive rate (*R*_0_), mean generation time (*T*), and intrinsic rate of increase (*r*) were calculated as specified in Table 1. The means and standard errors of the life table parameters were estimated using the bootstrap method with 100,000 iterations [23]. The TWOSEX-MSChart^®^ [24] software (Ver. 04/18/2024) was used to generate and analyze age-stage two-sex life tables. A built-in paired bootstrap test was used to compare differences between treatments [23,24,25]. The TIMING-MSChart^®^ [25] software (Ver. 05/07/2024) was used to predict population growth and structure of each age stage of *P. solenopsis* at different temperatures over a period of 90 days with an initial population of 10 eggs and without control.

## 3. Results

The developmental durations of different instars of *P. solenopsis* on tomatoes at considered temperatures are presented in Table 2. The increase in temperature caused a statistically significant decrease in the developmental periods of all instars except for eggs, as pointed out by the bootstrap test (*p* < 0.05). Overall, the longest total preadult duration was noted at 20 °C with 25.89 days for females and 22.8 days for males, while the shortest was 19.03 days for females and 3.27 days for males at 35 °C. Similarly, adult longevity was the highest at 20 °C (females: 3.15 days and males: 4.2 days) and the lowest at 35 °C (females: 19.03 days and males: 3.27 days). As a consequence, the longest duration of the life cycle of *P. solenopsis* was 59.05 days for females and 27 days for males at 20 °C, while the shortest was 29.58 days for females and 13.91 days for males at 35 °C.

Reproduction parameters of *P. solenopsis* on tomatoes at different temperatures are presented in Table 3. The adult preoviposition period and the total preoviposition period were reduced as a function of temperature increase, being the longest at 20 °C (APOP: 17.38 days and TPOP: 43.28 days) and the shortest at 35 °C (APOP: 7.78 days and TPOP: 18.33 days). Conversely, the number of oviposition days was the highest at 20 °C with 9.77 days, and the lowest at 35 °C with 8.05 days. Fecundity was also influenced by temperature as the highest was recorded at 30 °C (183.29 eggs/female), and the lowest was 113.35 eggs/female at 20 °C.

Population growth parameters of *P. solenopsis* on tomatoes at different temperatures are presented in Table 4. The average net reproduction rate (*R*_0_) of *P. solenopsis* significantly varied as a function of temperature. The highest value was obtained at 35 °C with 154.24 offspring/female, while the lowest was noted at 20 °C with 98.24 offspring/female. The intrinsic rate of increase (*r*) was the highest at 35 °C with 0.222 d^−1^ and the lowest at 20 °C with 0.101 d^−1^. The finite rate of increase (*λ*) was significantly higher at 35 °C 1.248 d^−1^ and the lowest at 20 °C 1.106 d^−1^. The generation time was the longest at 20 °C and shortest at 35 °C, with values of 45.142 days and 22.672 days, respectively.

**Figure 1:**
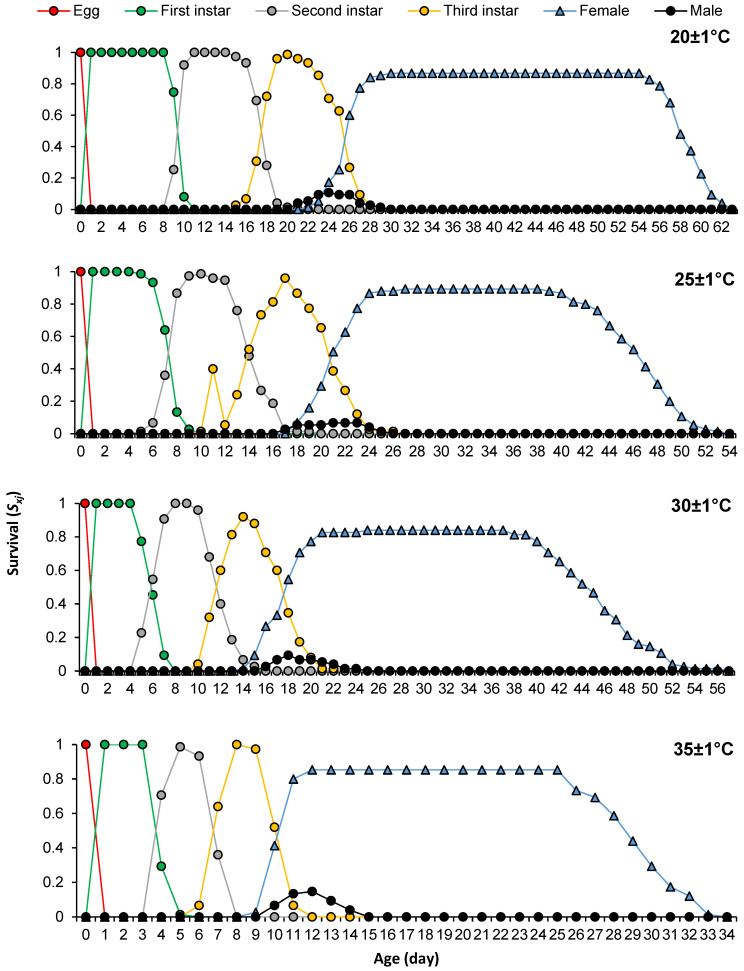
Survival rate of different developmental stages of *Phenacoccus solenopsis* on tomatoes at different temperatures (20 ± 1 °C, 25 ± 1 °C, 30 ± 1 °C and 35 ± 1 °C, 60 ± 5% RH, and 16:8 h (L:D)).

Curves describing the variation of age-stage-specific survival rate (*s_xj_*) of *P. solenopsis* on tomatoes at different temperatures are presented in Figure 1. The survival probabilities of preadult instars ranged between 0.92 and 1 at all tested temperatures, while those of females were 0.86, 0.89, 0.84, and 0.85, respectively, at 20 ± 1 °C, 25 ± 1 °C, 30 ± 1 °C, and 35 ± 1 °C. The lowest survival probability was recorded for males with 0.14 at all temperatures.

**Figure 2:**
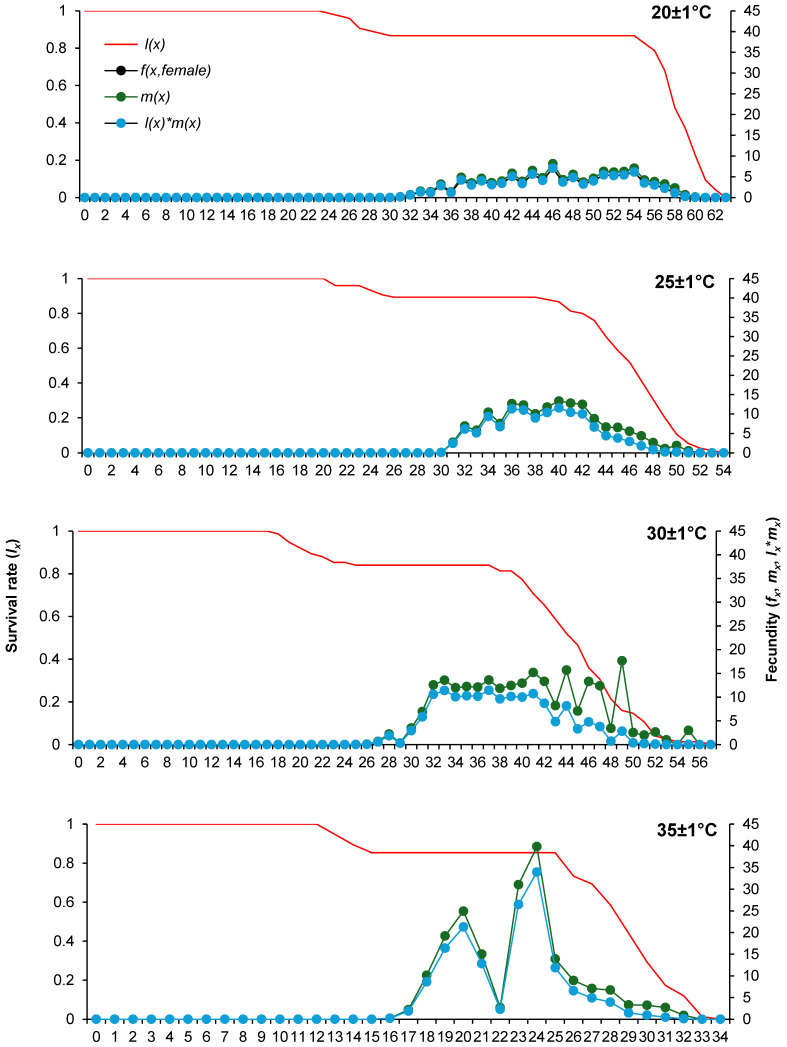
Age-specific survival rate (*l_x_*), female age-specific fecundity (*f_x_*), age-specific fecundity (*m_x_*) and age-specific maternity (l_x_*m_x_) versus age of *Phenacoccus solenopsis* on tomatoes at different temperatures (20 ± 1 °C, 25 ± 1 °C, 30 ± 1 °C and 35 ± 1 °C, 60 ± 5% RH, and 16:8 h (L:D)).

The age-specific survival rate (*l_x_*) was constantly between 80 and 100% during the preadult stages, which indicates a low mortality rate. This parameter started to decrease significantly on days 54, 39, 37, and 25, respectively, at 20 ± 1 °C, 25 ± 1 °C, 30 ± 1 °C, and 35 ± 1 °C. The age-stage-specific fecundity (*f_x_*) and the age-specific fecundity (*m_x_*) peaks were the greatest at 35 ± 1 °C and the lowest at 20 ± 1 °C (Figure 2).

**Figure 3:**
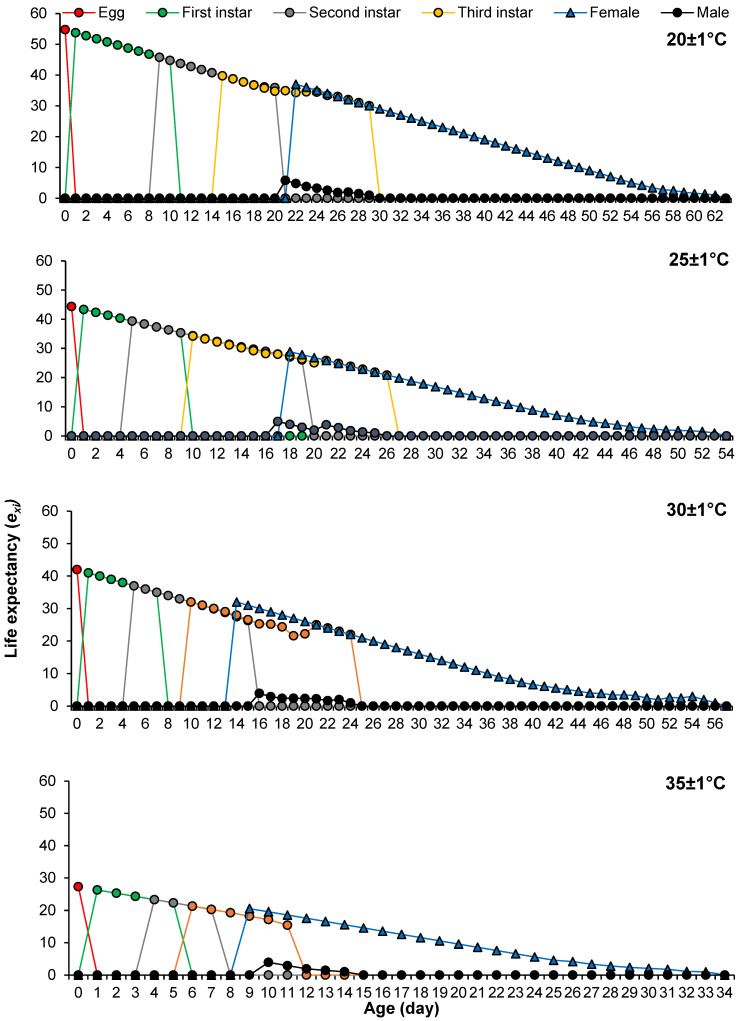
The age-stage life expectancy of *Phenacoccus solenopsis* on tomatoes at different temperatures (20 ± 1 °C, 25 ± 1 °C, 30 ± 1 °C and 35 ± 1 °C, 60 ± 5% RH, and 16:8 h (L:D)).

**Figure 4:**
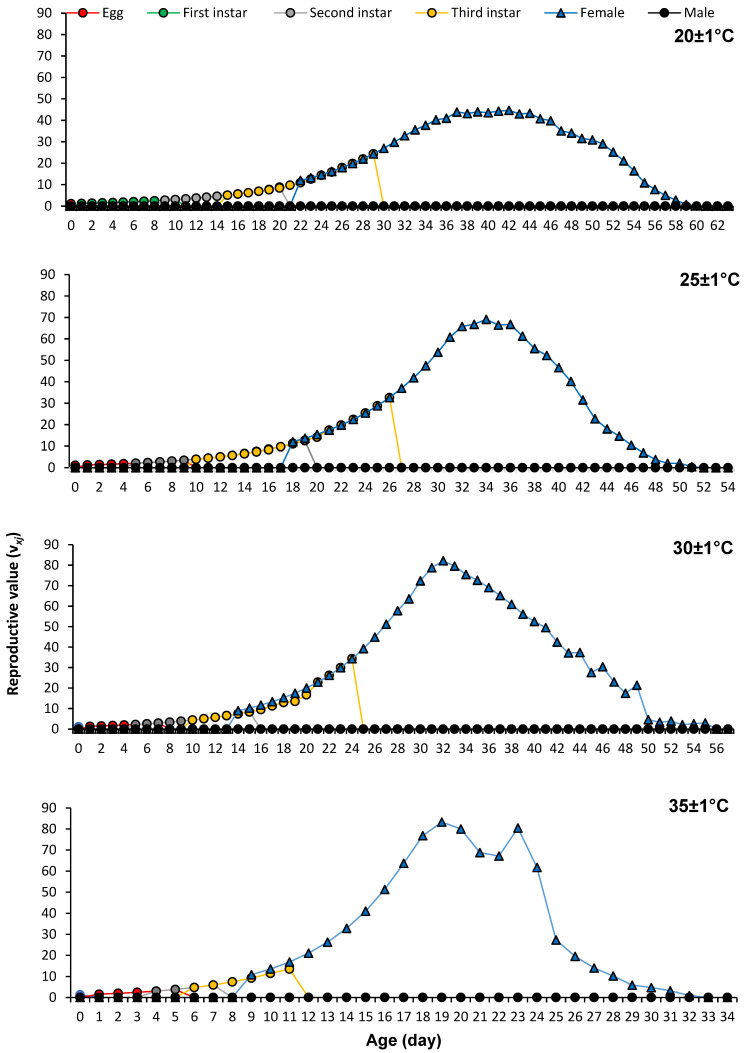
Age-stage reproductive values of *Phenacoccus solenopsis* on tomatoes at different temperatures (20 ± 1 °C, 25 ± 1 °C, 30 ± 1 °C and 35 ± 1 °C, 60 ± 5% RH, and 16:8 h (L:D)).

The age-stage life expectancy (*e_xj_*) of *P. solenopsis* decreased as temperature increased. At age zero, *e*^01^ was 53.77 days, 44.34 days, 41.98 days, and 27.28 days, respectively, at 20 ± 1 °C, 25 ± 1 °C, 30 ± 1 °C, and 35 ± 1 °C (Figure 3). The age-stage reproductive value (*v_xj_*) significantly increased as reproduction started, and *v_xj_* peaked on days 42, 34, 32, and 19 with values of 44.67 d^−1^, 69.12 d^−1^, 82.13 d^−1^, and 83.29 d^−1^ at 20 ± 1 °C, 25 ± 1 °C, 30 ± 1 °C, and 35 ± 1 °C, accordingly (Figure 4).

**Figure 5:**
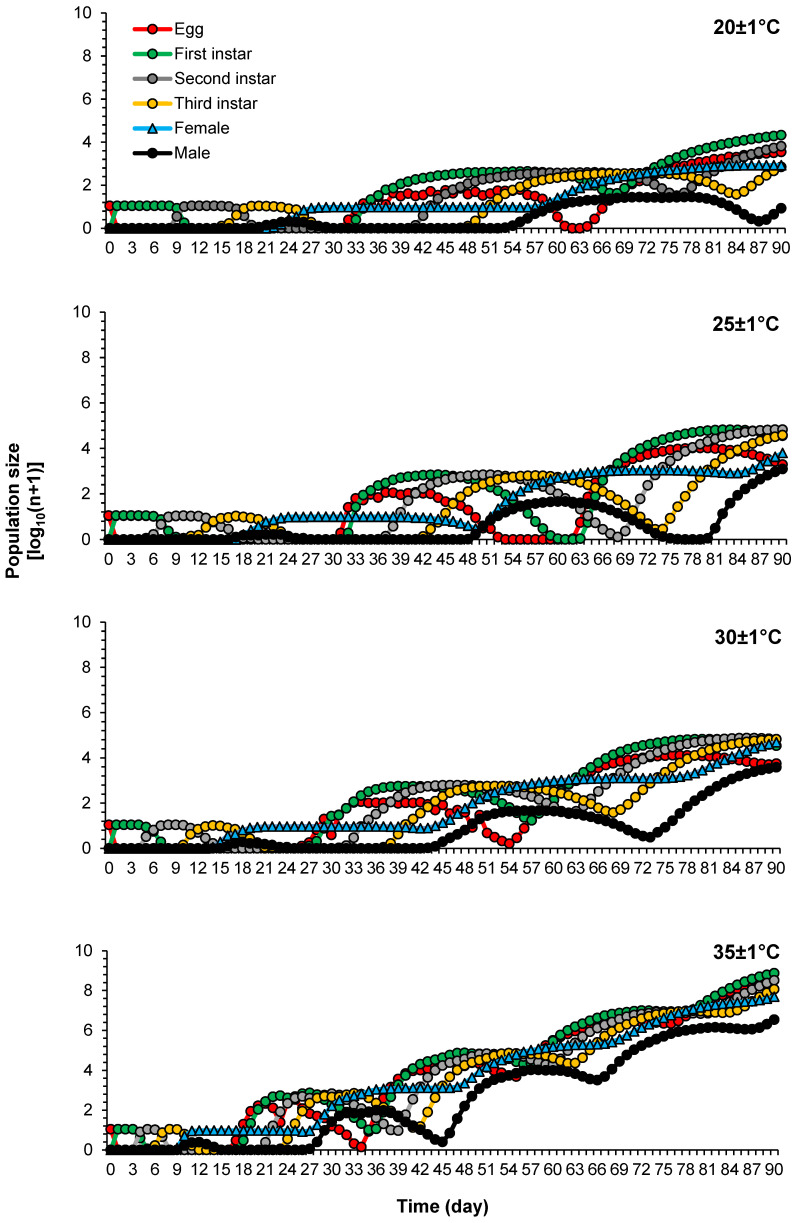
Population growth and structure predictions of *Phenacoccus solenopsis* on tomatoes at different temperatures (20 ± 1 °C, 25 ± 1 °C, 30 ± 1 °C and 35 ± 1 °C, 60 ± 5% RH and, 16:8 h (L:D)) starting from an initial population of 10 eggs and without control methods.

**Figure 6:**
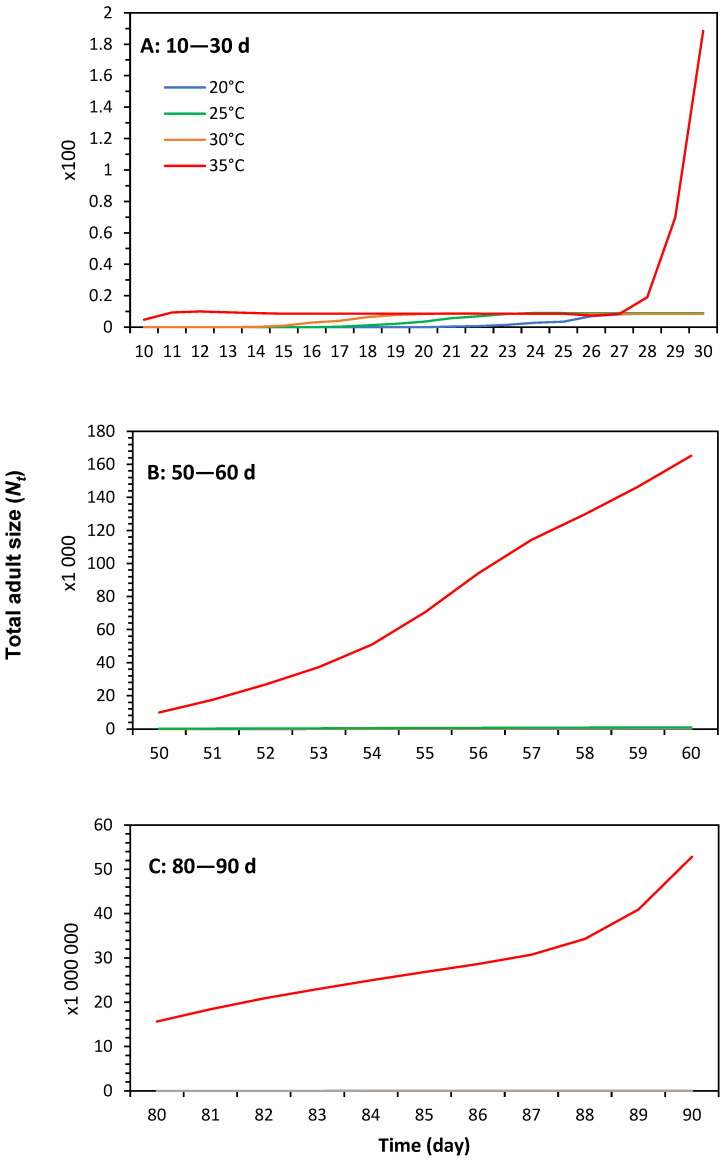
Total adult size (*N_t_*) of *Phenacoccus solenopsis* on tomatoes at different temperatures (20 ± 1 °C, 25 ± 1 °C, 30 ± 1 °C and 35 ± 1 °C, 60 ± 5% RH and 16:8 h (L:D)) in time intervals (**A**) 10–30 d, (**B**) 50–60 d, and (**C**) 80–90 d starting from an initial population of 10 eggs and without control methods.

Population growth parameters generated from life tables of *P. solenopsis* were used to simulate population increase and structure under different temperatures with the greatest expected population size at 35 °C. The projected number of generations increased with temperature and was 2, 2, 3, and 4 generations at 20 °C, 25 °C, 30 °C, and 35 °C, respectively (Figure 5). As a result of successive and overlapping generations, the total predicted adult size (*N_t_*) increased as a function of time (Figure 6) and was the highest at 35 °C, reaching nearly 53 million adults after a 90-day period without control (Figure 6C).

## 4. Discussion

*Phenacoccus solenopsis* has recently invaded the Mediterranean region. Tomato constitutes one of its main host plants in this area, and little work on the effect of temperature on its development, fecundity, and life history parameters on this crop has been carried out. Life tables provide valuable data on the life history parameters and the population growth trends of insects. Age-stage two-sex life tables include the roles of both females and males [21,25,30], which is crucial to accurately predict future population patterns and adapt control measures and the timing of their application.

Our results revealed a significant impact of temperature on this pest as high temperatures decreased the duration of the life cycle and the longevity of adults. Similarly, a shorter adult preoviposition period (APOP) and total preoviposition period (TPOP) were recorded at 35 °C. The highest fecundity was noted at 30 °C. This high temperature range allowed the highest net reproduction rate (*R_0_*), intrinsic rate of increase (*r*), and finite rate of increase (*λ*) and the shortest generation time (*T*).

Our data corroborate with those of Lu et al. [31] and Kumar and Kontodimas [32], who found that the full development of females and males of *P. solenopsis* on cotton was 414.9 and 218 degree days, correspondingly. They estimated that the lower temperature threshold for the total development of females is situated in the interval of 5.06–5.25 °C, and the optimum temperature is 33.55–33.60 °C, while the upper developmental threshold is 39.99–40 °C. Similarly, Fand et al. [33] concluded, through a modeling approach, that the lower development threshold temperature of *P. solenopsis* is 12.7 °C, the thermal constant is 97.1 degree days, and the optimum temperature range is 25–35 °C, allowing maximum fitness. Likewise, Kumar et al. [34] investigated the effects of temperature and relative humidity on the life table of *P. solenopsis* reared on cotton. They found that the fitness of the pest was the highest at 35 °C in combination with 65% RH.

Our projections of population increase and structure of *P. solenopsis* on tomatoes under different temperatures showed that the greatest population size is expected at 35 °C by allowing the completion of 4 generations in a 90-day period. Consequently, it can be stipulated that the temperature range of 30–35 °C guarantees maximum fitness and abundance of *P. solenopsis* on tomatoes. Although this temperature interval maximizes the fitness of the insect, it can also induce oxidative stress that can be overcome by increasing the activity of antioxidant enzymes to cope with oxidative cell damage, as demonstrated by Shankarganesh et al. [35].

Our results suggest that the most important infestations of Mediterranean tomato cultivations are expected to occur in protected and summer field crops where this range of temperatures prevails, with at least four generations per cropping cycle. Despite that, lower fitness parameters of *P. solenopsis* were recorded at the temperature range of 20–25 °C. Our data suggest that low to moderate infestations are likely to occur during the cold periods on early tomato crops conducted in greenhouses (from January to June) and in late tomato crops in open fields (from September to January), with less important population densities as pointed out by populations projections at 20 °C and 25 °C. However, other parameters, such as relative humidity, should also be considered, as proved by Kumar et al. [34], who found that the pest exhibited low biotic potential under a relative humidity of 75%; nymphs failed to complete their development at 85% RH. Such a situation can have severe consequences by reducing yields and disturbing already implemented IPM programs against other pests, such as the invasive South American tomato leafminer *Tuta absoluta* Meyrick (Lepidoptera: Gelechiidae). Consequently, adapted surveillance protocols and efficient control measures, both in open fields and greenhouses, are urgently needed to prevent potential outbreaks of *P. solenopis* in tomato crops in the Mediterranean region. Furthermore, it is crucial to consider the effects of the temperature on the potential distribution and damage of *P. solenopsis* on Mediterranean tomato crops since simulations expect a rise in its establishment probabilities, seasonal abundance, and damage worldwide under future climatic scenarios of global warming [33].

From another perspective, high temperatures can also indirectly impact population growth and dispersion of *P. solenopsis* by modulating the efficacy of its natural enemies. For instance, in the case of fortuitous coccinellid (Coleoptera: Coccinellidae) predators associated with this pest, it has been proven that the consumption rate of *Scymnus interruptus* Goeze and *Hippodamia convergens* Guérin-Ménevill on *P. solenopsis* was the lowest at 32 °C [36]. Conversely, *Nephus hiekei* Fursch and *Menochilus sexmaculatus* Fabricius exhibited the greatest performances at the same temperature range [37,38]. This was also the case for the spontaneous mealybug predator *Dicrodiplosis manihoti* Harris (Diptera: Cecidomiidae) [39]. For parasitoids, it has been shown, for example, that *Aenasius arizonensis* Girault (Hymenoptera: Encyrtidae), one of the most efficient natural enemies of *P. solenopsis* in India, exhibited maximum development and reproduction parameters at 30 ± 10 °C [40]. Accordingly, while maximizing the fitness of the pest, high temperatures can, in many cases, reduce the efficiency of its natural enemies, contributing to uncontrolled outbreaks. Consequently, the choice of natural enemies for the biological control of *P. Solenopsis* on tomato crops should consider their performance as a function of temperature as well as the fitness parameters of the pest to achieve efficient control.

## 5. Conclusions

Our study provides important information on the life history parameters of *P. Solenopsis*, an invasive pest threatening Mediterranean tomato cultivations. Generated life tables indicate that this pest can cause outbreaks in this crop in summer, particularly in greenhouses where temperature is optimal for its development. We also concluded that four generations could occur over a period of three months at 35 °C, which is a very important population size. Thus, it can be stipulated that this pest is likely to cause economic losses in Mediterranean tomato cultivations where summer is hot and winter is mild. Studying the effect of constant temperatures on the life history parameters of *P. Solenopsis* constitutes a first step for determining the effects of abiotic factors on its population dynamics on tomato crops under the Mediterranean climate. Nonetheless, further studies in real field conditions are needed for a better understanding of the biology of this new pest of tomatoes in the Mediterranean region. Data generated in this study can also be used to model pest dispersion and perform risk analyses.

## Figures and Tables

**Table 1:** Age-stage two-sex life table parameters considered to evaluate the fitness of *Phenacoccus solenopsis* on tomatoes at different temperatures (20 ± 1 °C, 25 ± 1 °C, 30 ± 1 °C and 35 ± 1 °C, 60 ± 5% RH, and 16:8 h (L:D)).

Parameters	Definition	Equation *	Reference
The age-specific survival rate (*l_x_*)	Probability that a newly laid egg will survive to age *x*	$l_{x}=\sum_{j=1}^{k}s_{xj}$	[21]
The age-specific fecundity (*m_x_*)	Mean fecundity of individuals at age *x*	$m_{x}=\frac{\sum_{j=1}^{k}s_{xj}f_{xj}}{\sum_{j=1}^{k}s_{xj}}$	[21]
The age-stage life expectancy (*e_xj_*)	Time that an individual of age *x* and stage *j* is expected to live	$e_{xj}=\sum_{i=x}^{\infty }\sum_{j=y}^{k}{s\prime }_{iy}$	[26,27]
The reproductive value (*v_xj_*)	Contribution of individuals of age *x* and stage *j* to the future population	$V_{xj}=\frac{e^{r(x+1)}}{s_{xj}}\sum_{i=x}^{\infty }e^{-r(i+1)}\sum_{j=y}^{k}s_{iy}^{\prime }f_{iy}$	[26,27]
The net reproductive rate (*R*_0_)	Total mean number of offspring that an average individual (including females, males, and those who died in the immature stage) can produce during their lifetime	$R_{0}=\sum_{x=0}^{\infty }l_{x}m_{x}$	[21,28]
The intrinsic rate of increase (*r*)	Number of deaths subtracted by the number of births per generation time	$r=\sum_{x=0}^{\infty }e^{-r(x+1)}l_{x}m_{x}=1$	[21,28]
The finite rate of increase (*λ*)	Population growth rate at a stable distribution	$\lambda =e^{r}$	[29]
The mean generation time (*T*)	Time required for a population to grow to *R*_0_-times its initial size at a stable distribution	$T=\frac{\ln R_{0}}{r}$	[21]

* Where *k* is the last stage of the study cohort, *n* is the number of age groups, *m* is the number of stages, and *s′_ij_* is the probability that an individual of age *x* and stage *j* will survive to age *i* and stage *y*.

**Table 2:** Developmental duration (day) and adult longevity (day) of *Phenacoccus solenopsis* on tomatoes at different temperatures (20 ± 1 °C, 25 ± 1 °C, 30 ± 1 °C and 35 ± 1 °C, 60 ± 5% RH and 16:8 h (L:D)).

	Temperatures			
Developmental Stage	20 ± 1 °C	25 ± 1 °C	30 ± 1 °C	35 ± 1 °C
Egg	1.00 ± 0.00 a (*n* = 75)	1.00 ± 0.00 a (*n* = 75)	1.00 ± 0.00 a (*n* = 75)	1.00 ± 0.00 a (*n* = 75)
First instar				
Female	8.83 ± 0.07 a (*n* = 42)	6.75 ± 0.11 b (*n* = 48)	5.17 ± 0.12 c (*n* = 41)	3.31 ± 0.06 d (*n* = 46)
Male	8.8 ± 0.20 a (*n* = 33)	6.50 ± 0.33 b (*n* = 27)	6.08 ± 0.15 b (*n* = 34)	3.27 ± 0.14 c (*n* = 29)
Second instar				
Female	8.14 ± 0.10 a (*n* = 41)	7.04 ± 0.19 b (*n* = 46)	5.94 ± 0.15 c (*n* = 39)	2.98 ± 0.06 d (*n* = 44)
Male	7.9 ± 0.23 a (*n* = 33)	5.75 ± 0.16 c (*n* = 26)	6.33 ± 0.38 b (*n* = 32)	3.00 ± 0.13 d (*n* = 29)
First instar				
Female	7.92 ± 0.07 a (*n* = 40)	6.55 ± 0.15 b (*n* = 46)	5.7 ± 0.20 c (*n* = 39)	3.25 ± 0.05 d (*n* = 43)
Male pupae	5.1 ± 0.38 b (*n* = 31)	6 ± 0.46 a (*n* = 25)	4.67 ± 0.14 c (*n* = 31)	3.36 ± 0.15 d (*n* = 28)
Total pre-adult				
Female	25.89 ± 0.18 a (*n* = 40)	21.34 ± 0.23 b (*n* = 46)	17.81 ± 0.25 c (*n* = 39)	10.55 ± 0.08 d (*n* = 43)
Male	22.8 ± 0.51 a (*n* = 31)	19.25 ± 0.67 b (*n* = 25)	18.08 ± 0.47 c (*n* = 31)	10.64 ± 0.2 d (*n* = 28)
Adult longevity				
Female	33.15 ± 0.16 a (*n* = 38)	25.51 ± 0.3 c (*n* = 44)	28.21 ± 0.43 b (*n* = 37)	19.03 ± 0.26 d (*n* = 41)
Male	4.20 ± 0.29 a (*n* = 29)	4.12 ± 0.35 a (*n* = 25)	2.75 ± 0.18 c (*n* = 28)	3.27 ± 0.14 b (*n* = 27)
Total life cycle				
Female	59.05 ± 0.25 a (*n* = 38)	46.85 ± 0.4 b (*n* = 44)	46.02 ± 0.52 b (*n* = 37)	29.58 ± 0.28 c (*n* = 41)
Male	27 ± 0.56 a (*n* = 29)	23.38 ± 0.73 b (*n* = 25)	20.83 ± 0.6 c (*n* = 28)	13.91 ± 0.25 d (*n* = 27)

Values are means ± standard errors. Means in a row followed by different letters are significantly different at *p* = 0.05 using a paired bootstrap test.

**Table 3:** Adult preoviposition period (APOP), total preoviposition period (TPOP), oviposition days and fecundity of *Phenacoccus solenopsis* on tomatoes at different temperatures (20 ± 1 °C, 25 ± 1 °C, 30 ± 1 °C and 35 ± 1 °C, 60 ± 5% RH and 16:8 h (L:D)).

Parameters	Temperatures			
	20 ± 1 °C	25 ± 1 °C	30 ± 1 °C	35 ± 1 °C
APOP (day)	17.38 ± 0.97 a	13.66 ± 0.14 c	15.71 ± 0.35 b	7.78 ± 0.09 d
TPOP (day)	43.28 ± 1 a	35.00 ± 0.28 b	33.52 ± 0.42 c	18.33 ± 0.13 d
Oviposition days (day)	9.77 ± 0.59 a	8.93 ± 0.35 b	9.37 ± 0.27 a	8.05 ± 0.25 c
Fecundity (eggs/female)	113.35 ± 7 d	139.27 ± 7.07 c	183.29 ± 7.13 a	180.75 ± 5.68 b

Values are means ± standard errors. Means in a row followed by different letters are significantly different at *p* = 0.05 using a paired bootstrap test.

**Table 4:** Net reproductive rate (*R*_0_), intrinsic rate of increase (*r*), finite rate of increase (*λ*) and generation time (*T*) of *Phenacoccus solenopsis* on tomatoes at different temperatures (20 ± 1 °C, 25 ± 1 °C, 30 ± 1 °C and 35 ± 1 °C, 60 ± 5% RH and 16:8 h (L:D)).

Parameters	Temperatures			
	20 ± 1 °C	25 ± 1 °C	30 ± 1 °C	35 ± 1 °C
*R*_0_ (offspring/female)	98.240 ± 11.231 c	124.413 ± 14.842 b	153.96 ± 15.325 a	154.24 ± 14.681 a
*r* (day^−1^)	0.101 ± 0.003 d	0.124 ± 0.002 c	0.134 ± 0.003 b	0.222 ± 0.003 a
*λ* (day^−1^)	1.106 ± 0.005 c	1.133 ± 0.004 b	1.143 ± 0.004 b	1.248 ± 0.004 a
*T* (day)	45.142 ± 0.553 a	38.616 ± 0.373 b	37.462 ± 0.574 c	22.672 ± 0.562 d

Values are means ± standard errors. Means in a row followed by different letters are significantly different at *p* = 0.05 using a paired bootstrap test.

## Data Availability

The data sets generated during and/or analyzed during the study are available from the corresponding author upon reasonable request.
